# Mechanism of Regulation of NaCl Homeostasis in the Distal Colon During Obesity

**DOI:** 10.3390/ijms26189139

**Published:** 2025-09-19

**Authors:** Balasubramanian Palaniappan, John Crutchley, Raja Singh Paulraj, Alip Borthakur, Subha Arthur

**Affiliations:** 1 Department of Biomedical Sciences, Joan C. Edwards School of Medicine, Marshall University, Huntington, WV 27501, USA; borthakur@marshall.edu (A.B.); arthursu@marshall.edu (S.A.); 2 Marshall Institute for Interdisciplinary Research, Marshall University, Huntington, WV 25703, USA; crutchleyj@marshall.edu; 3 Joan C. Edwards School of Medicine, Marshall University, Huntington, WV 27501, USA; paulraja@marshall.edu

**Keywords:** Cl^−^/HCO_3_^−^ exchanger, Downregulated in Adenoma (DRA), Na^+^/H^+^ exchanger, colon, obesity

## Abstract

Obesity is characterized by low-grade chronic inflammation, similar to the pathophysiology of inflammatory bowel disease (IBD) and colon cancer. IBD, which includes Crohn’s disease and ulcerative colitis, is becoming increasingly common in obese individuals. Our previous research documented that both IBD and obesity involve disrupted NaCl homeostasis in the small intestine. The present study investigated how obesity affects NaCl homeostasis in the distal colon, using the Zucker (*Lepr^fa^*) rat as a genetic model of obesity. The functional and molecular alterations in NaCl homeostasis were evaluated through radioactive uptakes, RT-qPCR, and Western blot studies. We found a significant reduction in Cl absorption via Cl^−^/HCO_3_^−^ exchanger, Downregulated in Adenoma (DRA) in the distal colon of obese rats compared to lean controls. This reduction was due to a decrease in the maximum transport capacity (V_max_) of DRA, with no change in the affinity of the exchanger for chloride. DRA mRNA and protein levels were also downregulated in obese animals. In contrast, Na absorption via Na^+^/H^+^ exchanger and its expression remained unchanged. These findings are the first to demonstrate that DRA is significantly impaired in the distal colon due to obesity. This suggests that net NaCl absorption in the distal colon is compromised in obesity, potentially increasing the risk for IBD and colon cancer.

## 1. Introduction

The global prevalence of obesity has reached epidemic levels, with nearly a three-fold increase since 1975. According to the World Health Organization, by 2050, an estimated 60% of males and 50% of females will be obese [1,2,3]. Obesity results from excessive body fat accumulation, driven by a combination of etiological factors such as high-calorie food consumption, sedentary lifestyles, obesogenic environmental influences, and genetic predispositions (mono and polygenic) [4,5,6]. Obesity significantly elevates the risk of numerous health complications, including type 2 diabetes, hypertension, metabolic syndrome, cardiovascular disease, inflammatory bowel disease (IBD), such as Crohn’s disease (CD) and Ulcerative colitis (UC), and colon cancers [7,8,9]. In obesity, disrupted glucose and NaCl homeostasis directly contribute to the pathogenesis of diabetes and hypertension [10]. Furthermore, electrolyte disturbances, though often underappreciated, play a pivotal role in metabolic dysfunction and systemic dysfunctions [11]. Therefore, elucidating the complex relationship between obesity and electrolyte disturbances is essential for developing comprehensive and effective treatment strategies.

The mammalian colon’s primary role is electrolyte (Na^+^ and Cl^−^) and water absorption. This is facilitated by the coupled activity of the Na^+^/H^+^ exchanger NHE3 (sodium: proton exchanger-3 encoded by the *SLC9A3* gene) and the Cl^−^/HCO_3_^−^ exchanger DRA (Downregulated in Adenoma encoded by the *SLC26A3* gene), with DRA predominantly expressed in the distal colon [12,13,14,15,16]. The regulation of electrolyte and acid/base transport by DRA and other transporters in colonic epithelial cells is vital for maintaining luminal pH, mucus secretion, and gut microbiome homeostasis, thereby preserving epithelial barrier integrity. Disruption of acid/base transport, especially reduced DRA expression, leads to increased intracellular alkalinity, a characteristic feature associated with the onset and progression of colitis-associated colon cancer (CAC) [17,18,19,20,21,22,23,24,25,26,27,28,29,30]. Obesity is a well-established risk factor and plays a crucial role in the early onset of colorectal cancer (CRC) [31]. Many studies have consistently documented the association between obesity and CRC. Further, loss of functional DRA leads to diarrheal diseases like inflammatory bowel disease (IBD) and congenital chloride diarrhea (CLD). Diarrhea, a key symptom in IBD, is also a prevalent symptom among obese patients and has been shown to result from disrupted intestinal epithelial transporter function that impacts fluid balance [32,33,34,35,36,37,38]. Moreover, there is a significant overlap between IBD and obesity, with 15–40% of IBD patients being overweight and 15–40% obese [39,40,41,42].

Our previous research demonstrated that in small intestinal epithelial cells from a monogenic rat model of obesity and obese humans, glucose and sodium absorption (via sodium-dependent glucose transporter, SGLT1) and chloride absorption via DRA are increased. This upregulation leads to enhanced glucose and NaCl absorption, providing a basis for the dysregulation of glucose and NaCl homeostasis observed in diabetes and hypertension associated with obesity [10]. Given DRA’s significant role in the intestine and the rising prevalence of IBD among obese individuals, it is crucial to explore whether DRA function is altered in the colon in the context of obesity. Since obesity is a known risk factor for colon cancer and DRA is involved in colon cancer development, understanding DRA regulation in the colon under obese conditions is essential. Therefore, this study aims to investigate DRA’s role and regulation in the colon in relation to obesity.

## 2. Results

### 2.1. Cl^−^/HCO_3_^−^ Exchange (DRA) Activity in the Colon During Obesity

**Figure 1:**
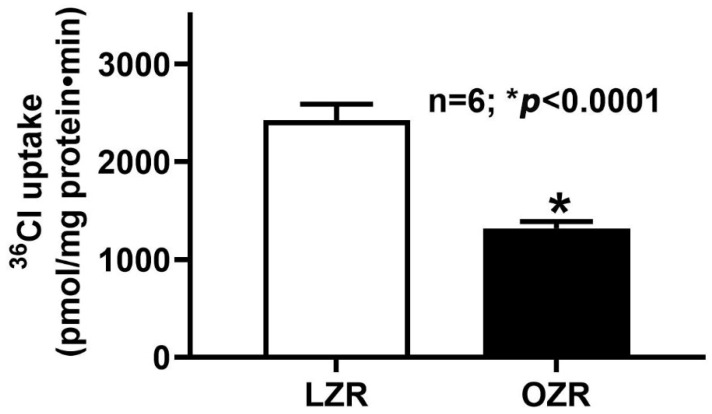
Cl^−^/HCO_3_^−^ exchange activity in the obese Zucker rat distal colon. DIDS (4,4’-diisothiocyanatostilbene-2,2′-disulfonic acid disodium salt)-sensitive HCO_3_^−^ dependent ^36^Cl^−^ uptake was significantly inhibited in the AMV of the distal colon of the obese Zucker rat compared with the lean (n = 6; * *p* < 0.0001). LZR: Lean Zucker Rat, OZR: Obese Zucker Rat, AMV: Apical Membrane Vesicle.

Lean and obese Zucker rat demonstrated Cl^−^/HCO_3_^−^ exchange activity in the distal colon (Figure 1). However, obese Zucker rats showed a significant reduction in Cl^−^/HCO_3_^−^ exchange activity (1315 ± 71.2 pmol/mg protein/min; n = 6; *p* < 0.0001) compared to lean Zucker rats (2424 ± 165.6 pmol/mg protein/min) (Figure 1). These results suggest that obesity impairs Cl^−^/HCO_3_^−^ exchange activity in the distal colon.

### 2.2. Na^+^/H^+^ Exchange (NHE3) Activity in the Distal Colon in Obesity

**Figure 2:**
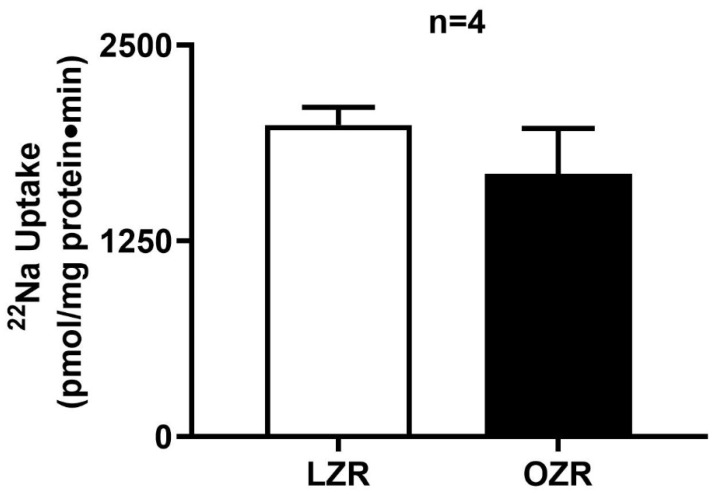
Na^+^/H^+^ exchange activity in the distal colon of the obese Zucker rat. Na^+^/H^+^ exchange activity was determined by pH-dependent amiloride-sensitive ^22^Na uptake, was comparable in the AMV of both the obese and lean Zucker rat distal colon (n = 4). This indicates Na^+^/H^+^ exchange was unaffected in obese animals. LZR: Lean Zucker Rat, OZR: Obese Zucker Rat, AMV: Apical Membrane Vesicle.

Na^+^/H^+^ exchange activity was observed in the distal colon AMVs of both lean and obese Zucker rats. However, as shown in Figure 2, Na^+^/H^+^ exchange activity remained unchanged between obese and lean Zucker rats (1678 ± 290.3 pmol/mg protein/min in obese Zucker rats; 1989 ± 113.9 in lean Zucker rats, n = 4).

Data shown in Figure 1 and Figure 2 together indicate that though sodium absorption is unaltered in obesity, Cl absorption is dysregulated, which indicates that the NaCl homeostasis is indeed affected in the distal colon in obesity.

### 2.3. Kinetic Studies of Cl^−^/HCO_3_^−^ Exchange in the Distal Colon in Obesity

**Figure 3:**
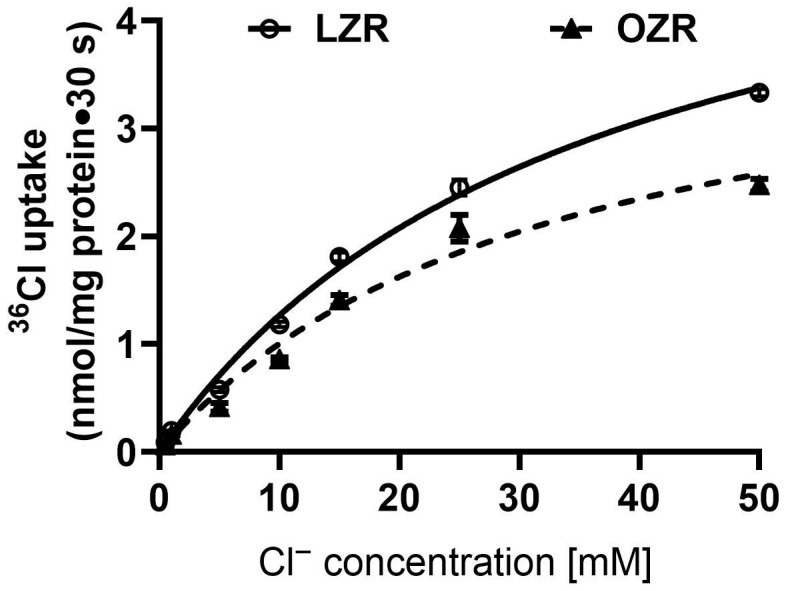
Kinetic studies of Cl^−^/HCO_3_^−^ exchange in the distal colon of the obese Zucker rat. ^36^Cl^−^ uptake at all concentrations was measured over 30 s, as the initial rate of uptake for Cl^−^/HCO_3_^−^ in AMV was linear for at least 60 s. As the extra vesicular chloride concentration increased, bicarbonate-dependent ^36^Cl^−^ uptake was also stimulated and then saturated under all conditions. The kinetic parameters from these studies are shown in Table 1; the maximal velocity (V_max_; nmol/mg protein · 30 s) is significantly reduced in the distal colonocytes of obese Zucker rats, whereas there is no change in the affinity of the exchangers (1/K_m_; mM) for chloride between the two groups. LZR: Lean Zucker rat; OZR: Obese Zucker rat (n = 3).

Kinetic studies were conducted to elucidate the mechanism of Cl^−^/HCO_3_^−^ exchange inhibition in the distal colon of obese Zucker rats. Figure 3 illustrates the extracellular concentration-dependent uptake of ^36^Cl^−^ at 30 s in both lean and obese rats. Kinetic parameters (Table 1) revealed no significant difference in the Michaelis constant (1/K_m_) for Cl^−^ uptake between obese (K_m_: 32.1 ± 5.7 mM) and lean (K_m_: 35.7 ± 3.2 mM) rats (n = 3), indicating no change in affinity. However, the maximal rate of uptake (V_max_) was significantly lower in obese rats (4.2 ± 0.4 nmol/mg protein/min) compared to lean rats (5.8 ± 0.3 nmol/mg protein/min; n = 3, *p* < 0.05). These results suggest that in the distal colon in obesity, inhibition of Cl^−^/HCO_3_^−^ exchange primarily occurs through a reduction in the maximal transport capacity (V_max_), without affecting the affinity of the exchanger for chloride.

### 2.4. RT-qPCR Analysis of DRA mRNA Expression in the Obese Zucker Rat

**Figure 4:**
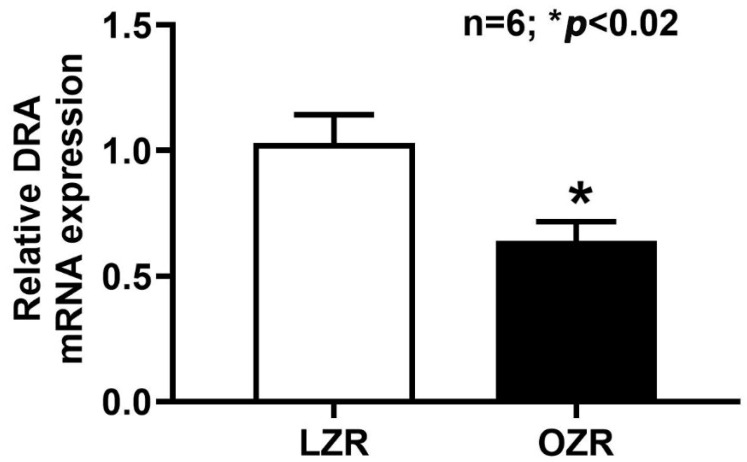
DRA mRNA levels in the distal colonocytes of the obese Zucker rats. DRA mRNA levels were present in both lean and obese Zucker rat distal colon. However, in obesity, DRA mRNA levels were moderately downregulated in the distal colon (n = 6, * *p* < 0.02).

To investigate the molecular basis for the observed reduction in Cl^−^/HCO_3_^−^ exchange, we examined DRA mRNA expression levels in distal colonocytes isolated from lean and obese Zucker rats using reverse transcription-quantitative polymerase chain reaction (RT-qPCR). The results, presented in Figure 4, demonstrate a decrease in DRA mRNA levels in the distal colon of obese Zucker rats compared to their lean counterparts. This finding suggests that the transcription of the DRA gene is downregulated, or reduced, in the distal colon of obese Zucker rats.

### 2.5. Western Blot Analysis

**Figure 5:**
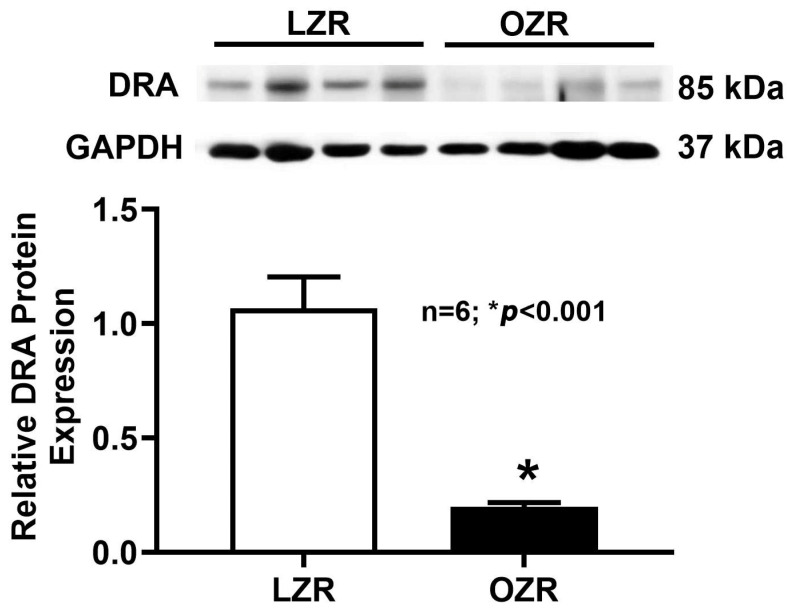
DRA protein expression in the distal colonocytes lysate of obese Zucker rats. Western blot studies revealed that the total cellular DRA protein expression of the distal colonocytes was significantly decreased in obese Zucker rats compared to lean rats (n = 6; * *p* < 0.001).

**Figure 6:**
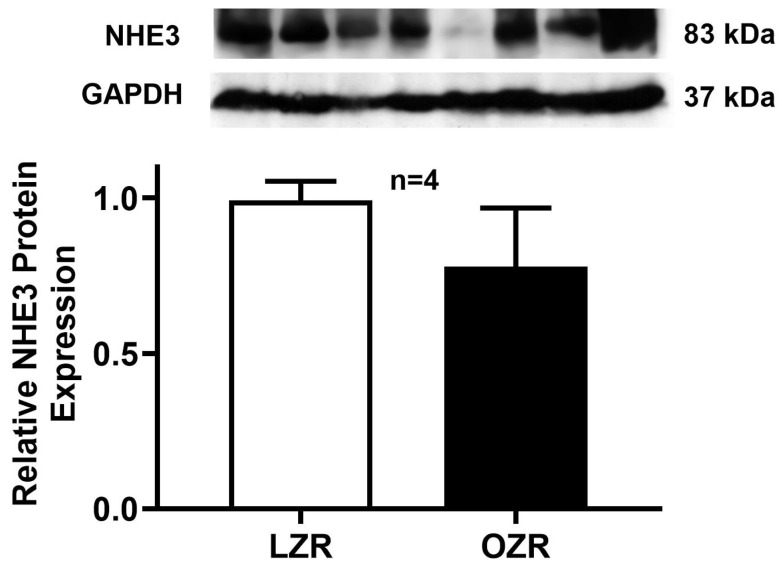
NHE3 protein expression in the distal colonocytes of the obese Zucker Rat. Western blot studies revealed that the total cellular NHE3 protein expression was unaltered between the distal colonocytes of obese and lean Zucker rats.

Although RT-qPCR analysis showed a decrease in DRA mRNA levels in obese rats, it is important to directly assess protein levels. Since the amount of mRNA does not always directly translate to the quantity of the functional protein. As shown in Figure 5, Western blot results for DRA protein expression in whole cell lysates revealed a significant decrease in immunoreactive DRA protein in the distal colonocytes of obese Zucker rats compared to lean Zucker rats. Moreover, consistent with our functional studies, NHE3 protein expression (Figure 6) remained unchanged between the lean and obese Zucker rat distal colonocytes. This finding confirmed that the observed reduction in DRA mRNA directly translates to a decrease in the total amount of DRA protein within the cells.

**Figure 7:**
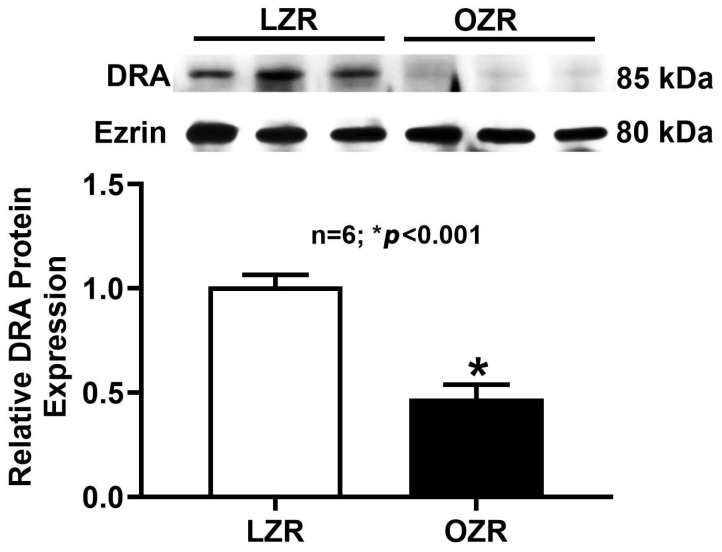
Apical membrane DRA protein expression in the distal colon of obese Zucker rats. Western blot studies revealed that the DRA protein expression in the apical membrane of the distal colonocytes from obese Zucker rats was significantly decreased compared to lean Zucker rats (n = 6; * *p* < 0.001).

We further examined DRA protein expression at the level of the AM, since AM is the cellular location where DRA facilitates its transport function. As shown in Figure 7, a decrease in AM-localized DRA protein was also observed in the distal colon of obese Zucker rats compared to lean Zucker rats. This suggests that the reduced cellular DRA protein is reflected in a decrease in the amount of DRA protein at the functional membrane surface.

## 3. Discussion

This study provides compelling new evidence that chloride/bicarbonate (Cl^−^/HCO_3_^−^) exchange activity mediated by DRA in the distal colon of obese Zucker rats is significantly diminished in obesity. Interestingly, Na^+^/H^+^ exchange (NHE3) functional activity and its expression in the distal colon remained unchanged between lean and obese animals. Our findings reveal that the mechanism underlying this obesity-associated inhibition in chloride absorption is due to a reduction in the number of functional Cl^−^/HCO_3_^−^ exchangers in the apical membrane of the colonocytes. Kinetic studies demonstrated that the affinity of the exchanger for chloride was unaltered, suggesting that the observed decrease in Cl^−^/HCO_3_^−^ exchange activity is not due to a change in the individual exchanger’s affinity properties, but a consequence of reduced DRA protein expression. This reduction, in turn, was due to the downregulation of de novo DRA mRNA synthesis in the distal colon of obese Zucker rats. Combining the total cellular and AM Western blot data, along with the kinetic parameters and RT-qPCR data, this study provides a comprehensive understanding of the mechanism underlying the inhibition of Cl^−^/HCO_3_^−^ exchange activity in the distal colon of obese Zucker rats and suggests that net NaCl absorption in the distal colon is impaired during obesity.

The etiology of obesity is complex and includes genetic factors that predispose an individual to gain weight very early in their life. The genetic causes of obesity include monogenic obesity that arises due to mutations in a single gene, specifically mutations in the leptin receptor. Patients with leptin receptor mutations experience an extremely early onset of obesity, which has a direct effect on hyperphagia, insulin sensitivity, glucose intolerance, metabolic disorders, and recurring infections [43]. Obese Zucker rats that mimic monogenic human obesity manifest similar clinical complications that have been reported in patients with leptin receptor mutations. Hence, the present study, designed with the obese Zucker rats, is an ideal model to study physiological alterations induced by obesity.

Obesity is a risk factor for gastrointestinal (GI) diseases [44]. Obesity is known to cause GI disease symptoms, including upper abdominal pain, gastroesophageal reflux, and diarrhea [45]. Population-based studies reveal that among the IBD patients, 15–40% are overweight and 20–40% are obese [39,40,41,42]. A study of the US adult population showed that chronic diarrhea correlates with obesity and that the risk of diarrhea increases with the severity of obesity [37,46]. The overall prevalence of chronic diarrhea among individuals with obesity in US population was 8.18% [32,37]. In obesity and IBD, disrupted NaCl homeostasis is a common symptom that directly contributes to the pathogenesis of hypertension and diarrhea [10,47].

NaCl homeostasis is maintained in the mammalian intestinal epithelial cells by the dual operation of the Na^+^/H^+^ exchanger NHE3 and the Cl^−^/HCO_3_^−^ exchanger DRA. The current study demonstrated that in obese Zucker rats, NaCl homeostasis in the distal colon is indeed dysregulated due to the downregulation of DRA and not NHE3. Two other chloride transporters in addition to DRA, namely Putative Anion Transporter-1 (PAT-1) and Cystic Fibrosis Transmembrane Conductance Regulator (CFTR) have also been implicated in the maintenance of NaCl homeostasis in the intestine. However, preliminary studies done in this Zucker model of obesity showed that the mRNA levels of these compensatory mechanisms were unchanged in the distal colon, indicating their nonparticipation in dysregulated NaCl homeostasis in the colon in obesity. Since this study shows that DRA is the only Cl transporter mechanism that is affected, loss of its function in the colon in obesity would suggest its clinical significance in disrupting luminal acid/base imbalance, causing defective mucus secretion and impaired epithelial barrier integrity, which would ultimately lead to diarrhea, a common symptom observed among IBD as well as in obesity patients. Research into diarrheal conditions such as IBD and CLD has consistently shown that altered DRA expression or mutations in the DRA gene result in decreased chloride absorption [48,49,50,51]. Furthermore, studies on DRA-deficient animals, which exhibit a phenotype similar to human CLD, confirm DRA’s crucial role in chloride absorption [14,17]. The dysregulation of DRA’s functional expression is also known to contribute to gut microbiome dysbiosis and disrupt mucosal immune homeostasis through epithelial-immune cell crosstalk, ultimately leading to IBD-related diarrheal diseases [13,16,52,53,54,55]. All these studies demonstrate the importance and implications of DRA in the pathophysiology of diarrheal diseases. The current study demonstrates that DRA is indeed a vital player in the pathophysiology of diarrhea, but among obese populations.

The data obtained from this study provides a different narrative compared to what was published earlier from the same animal model, but from a different intestinal region. In the small intestine, Na and glucose absorption via sodium-glucose co-transport (SGLT1) was found to be significantly increased. We also observed higher activity and expression of the DRA transporter in small intestinal villus cells, contradicting to our present findings in the colon in obesity. Notably, NHE3 activity and expression remained unchanged in the small intestine, indicating its non-participation in the increased Na and Cl absorption in the small intestine in obesity [10], similar to what was seen in the present study in the colon. These two studies indicate that DRA is differentially regulated along the intestine in obesity highlighting the significance of its dysregulation in the pathogenesis of obesity induced gut disorders that need to be explored in future studies.

As discussed earlier, obesity is a major risk factor for IBD and CRC. In addition, DRA expression is known to be downregulated in these conditions. The downregulation of DRA, as seen in this study, provides an important link between obesity and the higher incidence of CRC and IBD among obese patients. Moreover, CRC is the second-leading cause of cancer deaths worldwide and the most common obesity-related cancer in the US [56], and the downregulation of DRA in the colon appears to be a critical predisposing factor for the development of CRC among the obese population. Evidence shows that in IBD and CRC patients, mRNA and protein levels of DRA in the colonic apical membrane were decreased [21,27,57]. Many reports have implicated the role of proinflammatory cytokines (TNFα, INFγ, interleukins (ILs)), nitric oxide (NO), eicosanoids (arachidonic acid-PGE1,2, etc.), and their activated inflammatory signaling pathways (NF-κB, JAK-STAT, retinoic acid receptor-β/hepatocyte nuclear factor-1β, etc.), post-transcriptional (e.g., miRNA: miR-223-3p, miR-494) and post-translational regulators in the impaired DRA-mediated ion transport in inflamed colonic mucosa [58,59,60,61,62,63,64,65,66,67,68,69,70,71,72,73,74]. Multiple clinical and animal studies have found that proinflammatory immune cells are disturbed in obesity [75,76,77,78,79,80,81]. We speculate that inflammatory mediators and their downstream signaling pathways might be responsible for the deregulation of DRA-mediated electrolyte homeostasis in the obese distal colon. The schematic diagram below summarizes our current study.

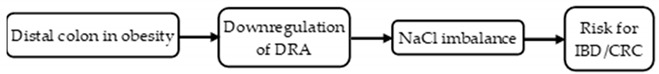



## 4. Materials and Methods

### 4.1. Chemicals

Except where indicated, all the chemicals used in this study were purchased from Sigma-Aldrich (Milwaukee, WI, USA).

### 4.2. Animal Models

Eighteen-week-old male obese (Lepr^fa^) Zucker rats (Strain 185) and lean Zucker rats (Strain 186) were obtained from Charles River Laboratories (Wilmington, MA, USA) as models of genetic obesity and lean controls, respectively. Animals were housed under a 12 h light/dark cycle with ad libitum access to food and water. The animal studies were performed in compliance with the ethical and procedural regulations of the Marshall University Institutional Animal Care and Use Committee (IACUC Protocol #777).

### 4.3. Colonocyte Isolation and Apical Membrane Vesicle Preparation

Distal colons were excised from lean and obese Zucker rats and rinsed twice with warm saline. Colonocytes were then isolated using a calcium chelation method [82]. Briefly, the distal colon was everted, filled with calcium-free Ringer solution (in mM: 127 NaCl, 10 HEPES, 5 KCl, 5 Na-pyruvate, 5 EDTA, 1 MgCl_2_, and 5 glucose; gassed with 95% O_2_ and 5% CO_2_, pH 7.4), and ligated at both ends. The colon was incubated in this solution at 37 °C for 20 min with gentle shaking to facilitate cell detachment. Following incubation, the colon was removed, and the resulting cell suspension was centrifuged at 4500× *g* for 2 min to pellet the colonocytes. Isolated colonocytes were immediately used for uptake studies or flash-frozen in liquid nitrogen and stored at −80 °C until needed. Apical membrane (AM) vesicles (AMVs) were prepared from stored colonocytes using the magnesium precipitation and differential centrifugation method, as previously described [83,84].

### 4.4. Uptake Studies in Intact Ileal Villus Cells and AMV

Chloride-bicarbonate (Cl^−^/HCO_3_^−^) exchange activity in AMVs was measured using a rapid-filtration technique, as previously described [10,67]. AMVs were suspended in a vesicle medium containing 5 mM N-methyl-D-glucamine (NMG) gluconate and 50 mM HEPES-Tris (pH 7.5), supplemented with either 100 mM KHCO_3_ (gassed with 5% CO_2_ and 95% N_2_) or 50 mM potassium gluconate (gassed with 100% N_2_). The reaction was initiated by adding 5 μL of AMV suspension to 95 μL of a reaction mixture containing 5 mM NMG ^36^Cl^−^ (American Radiolabeled Chemicals, St. Louis, MO, USA) 15 mM potassium gluconate, and 50 mM MES-Tris (pH 5.5), with or without 1 mM 4,4′-diisothiocyanatostilbene-2,2′-disulfonic acid disodium salt (DIDS), a Cl^−^/HCO_3_^−^ exchange inhibitor. The reaction was terminated after 60 s by adding 5 mL of ice-cold stop solution (50 mM HEPES-Tris, pH 7.5, 0.10 mM MgSO_4_, 50 mM potassium gluconate, and 100 mM NMG gluconate). The mixture was then filtered through 0.45 μm Millipore (HAWP) filter and washed twice with 5 mL of ice-cold stop solution. Filters were dissolved in 4 mL of scintillation fluid (Ecoscint; National Diagnostics, Atlanta, GA, USA), and radioactivity was measured using a Perkin Elmer Tri-Carb LSC 4910TR Scintillation Counter. Cl^−^/HCO_3_^−^ exchange activity was calculated as the DIDS-sensitive, HCO_3_^−^ dependent [^36^Cl^−^] uptake.

Kinetic parameters for Cl^−^/HCO_3_^−^ exchange were determined using intact colonocytes isolated from lean and obese Zucker rats. Briefly, 100 mg of isolated colonocytes were suspended in a buffer containing 5 mM NMG gluconate and 50 mM HEPES-Tris (pH 7.5), supplemented with either 100 mM KHCO_3_ or 100 mM potassium gluconate. The reaction was initiated by adding 10 μL of colonocyte suspension to 90 μL of a reaction medium containing 5 mM NMG [^36^Cl^−^], 150 mM potassium gluconate, 50 mM MES-Tris (pH 5.5), and varying concentrations of HCl (0.5, 1, 5, 15, 25, and 50 mM), with or without 1 mM DIDS. After a 30 s incubation, the reaction was terminated by adding an ice-cold stop solution (50 mM HEPES-Tris, pH 7.5, 0.10 mM MgSO_4_, 50 mM potassium gluconate, and 100 mM NMG gluconate). The mixture was then filtered through 0.65 μm Millipore (MCE MilliporeSigma, Burlington, MA, USA) filters and processed as described above. Michaelis-Menten kinetics were determined using nonlinear regression analysis of the uptake data in GraphPad Prism 9 (GraphPad Software, La Jolla, CA, USA).

For Na^+^/H^+^ exchange activity studies, AMV of the distal colon was suspended in a vesicle medium consisting of either 50 mM Tris-MES (pH 5.5) or 50 mM Tris-HEPES (pH 7.5), along with 300 mM mannitol. A 5 μL aliquot of this suspension was then incubated in a 95 μL reaction medium (300 mM mannitol, 50 mM Tris-HEPES (pH 7.5), and 1 mM ^22^Na^+^ (Eckert & Ziegler, Atlanta, GA, USA), with or without 1 mM amiloride. The uptake was terminated after 60 s by adding an ice-cold stop solution containing 300 mM mannitol and 50 mM Tris-HEPES (pH 7.5). This was followed by a rapid filtration process and radioactivity determination as described above. Na^+^/H^+^ exchange activity was calculated as the amiloride-sensitive, pH-dependent [^22^Na^+^] uptake.

### 4.5. Real-Time Quantitative PCR (RT-qPCR) for DRA

Total RNA was isolated from colonocytes of lean and obese Zucker rats using the RNeasy Mini Kit (Qiagen, Germantown, MD, USA) according to the manufacturer’s protocol. The RNA was reverse-transcribed and amplified with a Brilliant SYBR Green RT-qPCR Master Mix Kit (Qiagen One-Step RT-PCR Kit: Ref:210212, Germantown, MD, USA). Rat DRA was amplified using gene-specific primers custom-designed by Thermo Fisher Scientific (Hillsboro, OR, USA), with GAPDH amplified as an internal control. The sequences of the rat primers used in this study are as follows: DRA: forward primer: TACTGTCTCCCAGAACAGGACT; reverse primer: CGCTTCTTTCTGGCTGTTAGC, and for GAPDH: forward: AGGTCGGTGTGAACGGATTT and reverse: CCACTTTGTCACAAGAGAAGGC.

### 4.6. Western Blotting

Western blot analyses were performed on whole cell and AM protein extracts from lean and obese Zucker rat colonocytes, as previously described [85]. Proteins were extracted using RIPA buffer (50 mM Tris-HCl pH 7.4, 1% Igepal, 150 mM NaCl, 1 mM EDTA, 1 mM PMSF, 1 mM Na_3_VO_4_, 1 mM NaF) supplemented with protease inhibitor cocktail (SAFC Biosciences, Lenexa, KS, USA). The extracted protein was mixed with sample buffer and separated on 8% (custom-made) polyacrylamide gels. Separated proteins were then transferred to BioTrace PVDF membranes, blocked, and incubated overnight at 4 °C with the following primary antibodies (1:1000 dilution): mouse anti-DRA (SC-376187, Santa Cruz Biotechnology, Inc., Dallas, TX, USA), rabbit anti-Ezrin (ab231907, Abcam, Waltham, MA, USA) for AM, and rabbit anti-GAPDH for whole cell lysates. Membranes were then incubated with horseradish peroxidase-conjugated secondary antibodies (1:10,000 dilution): goat anti-mouse IgG (1706516, Bio-Rad Laboratories, 2000 Alfred Nobel Dr., Hercules, CA, USA) for DRA and mouse anti-rabbit IgG (sc-2357, Santa Cruz, CA, USA) for Ezrin and GAPDH, for 1 h at room temperature. Chemiluminescence was detected using ECL Detection Reagent (GE Healthcare, Chicago, IL, USA) and autoradiography. DRA protein density was quantified using ImageJ software J 1.53 t.

### 4.7. Protein Estimation

Protein quantification for both uptakes and Western blot studies was performed using the DCTM protein assay kit (Bio-Rad, Berkeley, CA, USA) following the Lowry method, according to the manufacturer’s protocol.

### 4.8. Statistical Analysis

All results are presented as means ± SEM, calculated using GraphPad Prism 9. The ‘n’ value represents the number of independent experiments, with each experiment performed using cells isolated from a different animal. For uptake experiments, each ‘n’ also indicates the average of triplicate measurements. Statistical significance was determined using a two-tailed Student’s *t*-test in GraphPad Prism 9. A *p*-value of <0.05 was considered statistically significant.

## 5. Conclusions

Our study shows that DRA is downregulated in the distal colon during obesity, in contrast to what was seen in the small intestine, highlighting regional differences in DRA regulation in the gut during obesity. This downregulation in the colon may contribute directly or indirectly to early CRC development, beyond just NaCl imbalance. Therefore, further research is needed to define and understand the consequences of DRA downregulation in the colon in obesity in the context of the pathophysiology of obesity-associated colon disorders such as colorectal cancer.

## Figures and Tables

**Table 1:** Kinetic parameters of Cl^−^/HCO_3_^−^ exchange in the distal colonocytes of obese Zucker rats.

	*K_m_* (mM)	*V_max_* (nmol/mg Protein·30 s)
Lean Zucker rat	35.7 ± 3.2	5.8 ± 0.3
Obese Zucker rat	32.1 ± 5.7	4.2 ± 0.4 *

n = 3, * *p* < 0.05

## Data Availability

The authors confirm that the data supporting the findings of this study are available within the article.

## Abbreviations

IBD	Inflammatory Bowel Disease
CD	Crohn’s Disease
UC	Ulcerative colitis
NaCl	Sodium chloride
DRA	Downregulated in Adenoma
NHE3	Sodium proton exchanger 3
SGLT1	Sodium-dependent glucose co-transport 1
CFTR	Cystic Fibrosis Transmembrane Conductance Regulator
PAT-1	Putative Anion Transporter-1
CAC	Colitis-associated colon cancer
CRC	Colorectal cancer
CLD	Congenital chloride diarrhea
DIDS	4,4′-diisothiocyanatostilbene-2,2′-disulfonic acid disodium salt
LZR	Lean Zucker Rat
OZR	Obese Zucker Rat
AMV	Apical membrane vesicle
GI	Gastrointestinal
TNFα	Tumor Necrosis Factor alpha
INFγ	Interferon gamma
IL	Interleukins
NO	Nitric oxide
NF-kB	Nuclear factor kappa B
miRNA	micro ribonucleic acid

## References

[1] Blüher M. (2019). Obesity: Global epidemiology and pathogenesis. Nat. Rev. Endocrinol..

[2] Koliaki C., Dalamaga M., Liatis S. (2023). Update on the Obesity Epidemic: After the Sudden Rise, Is the Upward Trajectory Beginning to Flatten?. Curr. Obes. Rep..

[3] Ward Z.J., Bleich S.N., Cradock A.L., Barrett J.L., Giles C.M., Flax C., Long M.W., Gortmaker S.L. (2019). Projected US state-level prevalence of adult obesity and severe obesity. N. Engl. J. Med..

[4] Khera A.V., Chaffin M., Wade K.H., Zahid S., Brancale J., Xia R., Distefano M., Senol-Cosar O., Haas M.E., Bick A. (2019). Polygenic prediction of weight and obesity trajectories from birth to adulthood. Cell.

[5] Farooqi I.S. (2022). Monogenic obesity syndromes provide insights into the hypothalamic regulation of appetite and associated behaviors. Biol. Psychiatry.

[6] Loos R.J., Yeo G.S. (2022). The genetics of obesity: From discovery to biology. Nat. Rev. Genet..

[7] Kopelman P.G. (2000). Obesity as a medical problem. Nature.

[8] Tarasiuk A., Mosińska P., Fichna J. (2018). The mechanisms linking obesity to colon cancer: An overview. Obes. Res. Clin. Pract..

[9] Gibson T.M., Park Y., Robien K., Shiels M.S., Black A., Sampson J.N., Purdue M.P., Freeman L.E.B., Andreotti G., Weinstein S.J. (2014). Body mass index and risk of second obesity-associated cancers after colorectal cancer: A pooled analysis of prospective cohort studies. J. Clin. Oncol..

[10] Palaniappan B., Arthur S., Sundaram V.L., Butts M., Sundaram S., Mani K., Singh S., Nepal N., Sundaram U. (2019). Inhibition of intestinal villus cell Na/K-ATPase mediates altered glucose and NaCl absorption in obesity-associated diabetes and hypertension. FASEB J. Off. Publ. Fed. Am. Soc. Exp. Biol..

[11] Crintea I.N., Cindrea A.C., Mederle O.A., Trebuian C.I., Timar R. (2025). Electrolyte Imbalances and Metabolic Emergencies in Obesity: Mechanisms and Clinical Implications. Diseases.

[12] Alper S.L., Sharma A.K. (2013). The SLC26 gene family of anion transporters and channels. Mol. Asp. Med..

[13] Priyamvada S., Gomes R., Gill R.K., Saksena S., Alrefai W.A., Dudeja P.K. (2015). Mechanisms Underlying Dysregulation of Electrolyte Absorption in Inflammatory Bowel Disease–Associated Diarrhea. Inflamm. Bowel Dis..

[14] Ishiguro H. (2014). HCO_3_^−^ secretion by SLC 26A3 and mucosal defence in the colon. Acta Physiol..

[15] Talbot C., Lytle C. (2010). Segregation of Na/H exchanger-3 and Cl/HCO3 exchanger SLC26A3 (DRA) in rodent cecum and colon. Am. J. Physiol.-Gastrointest. Liver Physiol..

[16] Hayashi H., Nagai H., Ohba K.-I., Soleimani M., Suzuki Y. (2021). Segmental differences in Slc26a3-dependent Cl^−^ absorption and HCO_3_^−^ secretion in the mouse large intestine in vitro in Ussing chambers. J. Physiol. Sci..

[17] Grivennikov S.I. (2013). Inflammation and colorectal cancer: Colitis-associated neoplasia. Semin. Immunopathol..

[18] Simpson J.E., Schweinfest C.W., Shull G.E., Gawenis L.R., Walker N.M., Boyle K.T., Soleimani M., Clarke L.L. (2007). PAT-1 (Slc26a6) is the predominant apical membrane Cl^−^/HCO_3_^−^ exchanger in the upper villous epithelium of the murine duodenum. Am. J. Physiol. Gastrointest. Liver Physiol..

[19] Xiao F., Yu Q., Li J., Johansson M., Singh A., Xia W., Riederer B., Engelhardt R., Montrose M., Soleimani M. (2014). Slc26a3 deficiency is associated with loss of colonic HCO_3_^−^ secretion, absence of a firm mucus layer and barrier impairment in mice. Acta Physiol..

[20] Webb B.A., Chimenti M., Jacobson M.P., Barber D.L. (2011). Dysregulated pH: A perfect storm for cancer progression. Nat. Rev. Cancer.

[21] Yang H., Jiang W., Furth E.E., Wen X., Katz J.P., Sellon R.K., Silberg D.G., Antalis T.M., Schweinfest C.W., Wu G.D. (1998). Intestinal inflammation reduces expression of DRA, a transporter responsible for congenital chloride diarrhea. Am. J. Physiol. Gastrointest. Liver Physiol..

[22] Lohi H., Makela S., Pulkkinen K., Hoglund P., Karjalainen-Lindsberg M.-L., Puolakkainen P., Kere J. (2002). Upregulation of CFTR expression but not SLC26A3 and SLC9A3 in ulcerative colitis. Am. J. Physiol. Gastrointest. Liver Physiol..

[23] Asano K., Matsushita T., Umeno J., Hosono N., Takahashi A., Kawaguchi T., Matsumoto T., Matsui T., Kakuta Y., Kinouchi Y. (2009). A genome-wide association study identifies three new susceptibility loci for ulcerative colitis in the Japanese population. Nat. Genet..

[24] Schweinfest C.W., Henderson K.W., Suster S., Kondoh N., Papas T.S. (1993). Identification of a colon mucosa gene that is down-regulated in colon adenomas and adenocarcinomas. Proc. Natl. Acad. Sci. USA.

[25] Antalis T.M., Reeder J.A., Gotley D.C., Byeon M.K., Walsh M.D., Henderson K.W., Papas T.S., Schweinfest C.W. (1998). Down-regulation of the down-regulated in adenoma (DRA) gene correlates with colon tumor progression. Clin. Cancer Res..

[26] Zhang N., Heruth D.P., Wu W., Zhang L.Q., Nsumu M.N., Shortt K., Li K., Jiang X., Wang B., Friesen C. (2019). Functional characterization of SLC26A3 c. 392C>G (p. P131R) mutation in intestinal barrier function using CRISPR/CAS9-created cell models. Cell Biosci..

[27] Arthur S., Palaniappan B., Sundaram U. (2013). Mo1798 Unique Regulation of Apical Membrane Cl: HCO_3_ Exchange (DRA) in Chronic Colitis Associated Cancer in Rats. Gastroenterology.

[28] Rubin D.C., Shaker A., Levin M.S. (2012). Chronic intestinal inflammation: Inflammatory bowel disease and colitis-associated colon cancer. Front. Immunol..

[29] Eaden J., Abrams K., Mayberry J. (2001). The risk of colorectal cancer in ulcerative colitis: A meta-analysis. Gut.

[30] Canavan C., Abrams K., Mayberry J. (2006). Meta-analysis: Colorectal and small bowel cancer risk in patients with Crohn’s disease. Aliment. Pharmacol. Ther..

[31] Xu P., Tao Z., Yang H., Zhang C. (2024). Obesity and early-onset colorectal cancer risk: Emerging clinical evidence and biological mechanisms. Front. Oncol..

[32] Han K., Wang X., Niu X., Li T., Linghu E. (2024). Prevalence and associated factors of chronic diarrhea among adults with obesity in the United States: Evidence from the National Health and Nutrition Examination Survey 2005 to 2010. Obes. Res. Clin. Pract..

[33] Han K., Wang X., Wang Y., Niu X., Xiang J., Ru N., Jia C., Sun H., He Z., Feng Y. (2024). Prevalence of chronic diarrhea and its association with obesity in a Chinese community-based population. China Med. J..

[34] Hihnala S., Höglund P., Lammi L., Kokkonen J., Örmälä T., Holmberg C. (2006). Long-term clinical outcome in patients with congenital chloride diarrhea. J. Pediatr. Gastroenterol. Nutr..

[35] McCoy J., Miller M.R., Watson M., Crowley E., Woolfson J.P. (2024). Paediatric obesity and Crohn’s disease: A descriptive review of disease phenotype and clinical course. Paediatr. Child Health.

[36] Zhuang Y., Li L., Sun J., Zhang Y., Dai F. (2025). Association of body roundness index with chronic diarrhea and constipation, NHANES 2005–2010. J. Health Popul. Nutr..

[37] Ballou S., Singh P., Rangan V., Iturrino J., Nee J., Lembo A. (2019). Obesity is associated with significantly increased risk for diarrhoea after controlling for demographic, dietary and medical factors: A cross-sectional analysis of the 2009–2010 National Health and Nutrition Examination Survey. Aliment. Pharmacol. Ther..

[38] Kobayashi M., Pattarathitwat P., Pongprajakand A., Kongkaew S. (2023). Association of normal weight obesity with lifestyle and dietary habits in young Thai women: A cross-sectional study. Obes. Pillars.

[39] Khakoo N.S., Ioannou S., Khakoo N.S., Vedantam S., Pearlman M. (2022). Impact of Obesity on Inflammatory Bowel Disease. Curr. Gastroenterol. Rep..

[40] Nguyen N.H., Ohno-Machado L., Sandborn W.J., Singh S. (2019). Obesity is independently associated with higher annual burden and costs of hospitalization in patients with inflammatory bowel diseases. Clin. Gastroenterol. Hepatol..

[41] Johnson A.M., Loftus E.V. (2020). Impact of obesity on the management of inflammatory bowel disease. Gastroenterol. Hepatol..

[42] Singh S., Dulai P.S., Zarrinpar A., Ramamoorthy S., Sandborn W.J. (2017). Obesity in IBD: Epidemiology, pathogenesis, disease course and treatment outcomes. Nat. Rev. Gastroenterol. Hepatol..

[43] Nunziata A., Funcke J.-B., Borck G., Von Schnurbein J., Brandt S., Lennerz B., Moepps B., Gierschik P., Fischer-Posovszky P., Wabitsch M. (2019). Functional and phenotypic characteristics of human leptin receptor mutations. J. Endocr. Soc..

[44] Camilleri M., Malhi H., Acosta A. (2017). Gastrointestinal complications of obesity. Gastroenterology.

[45] Eslick G. (2012). Gastrointestinal symptoms and obesity: A meta-analysis. Obes. Rev..

[46] Ciccocioppo R., De Giorgio R. (2020). Letter: Diarrhoea in obese patients-a new nosographic entity?. Aliment. Pharmacol. Ther..

[47] Anbazhagan A.N., Priyamvada S., Alrefai W.A., Dudeja P.K. (2018). Pathophysiology of IBD associated diarrhea. Tissue Barriers.

[48] Kere J. (2006). Overview of the SLC26 family and associated diseases. Proceedings of the Novartis Foundation Symposium 273, Epithelial Anion Transport in Health and Disease: The Role of the SLC26 Transporters Family.

[49] Kere J., Lohi H., Höglund P. (1999). Genetic Disorders of Membrane Transport III. Congenital chloride diarrhea. Am. J. Physiol..

[50] Mäkelä S., Kere J., Holmberg C., Höglund P. (2002). SLC26A3 mutations in congenital chloride diarrhea. Hum. Mutat..

[51] Schweinfest C.W., Spyropoulos D.D., Henderson K.W., Kim J.H., Chapman J.M., Barone S., Worrell R.T., Wang Z., Soleimani M. (2006). slc26a3 (dra)-deficient mice display chloride-losing diarrhea, enhanced colonic proliferation, and distinct up-regulation of ion transporters in the colon. J. Biol. Chem..

[52] Jayawardena D., Priyamvada S., Kageyama T., White Z., Kumar A., Griggs T.F., Majumder A., Akram R., Anbazhagan A.N., Sano T. (2023). Loss of SLC26A3 Results in Colonic Mucosal Immune Dysregulation via Epithelial-Immune Cell Crosstalk. Cell. Mol. Gastroenterol. Hepatol..

[53] Kumar A., Jayawardena D., Priyamvada S., Anbazhagan A.N., Chatterjee I., Saksena S., Dudeja P.K. (2024). SLC26A3 (DRA, the Congenital Chloride Diarrhea Gene): A Novel Therapeutic Target for Diarrheal Diseases. Cell. Mol. Gastroenterol. Hepatol..

[54] Kumar A., Priyamvada S., Ge Y., Jayawardena D., Singhal M., Anbazhagan A.N., Chatterjee I., Dayal A., Patel M., Zadeh K. (2021). A novel role of SLC26A3 in the maintenance of intestinal epithelial barrier integrity. Gastroenterology.

[55] Yu Q. (2021). Slc26a3 (DRA) in the gut: Expression, function, regulation, role in infectious diarrhea and inflammatory bowel disease. Inflamm. Bowel Dis..

[56] Bray F., Ferlay J., Soerjomataram I., Siegel R., Torre L., Jemal A. (2020). Erratum: Global cancer statistics 2018: GLOBOCAN estimates of incidence and mortality worldwide for 36 cancers in 185 countries. CA A Cancer J. Clin..

[57] Hosseini S.T., Nemati F. (2023). Identification of GUCA2A and COL3A1 as prognostic biomarkers in colorectal cancer by integrating analysis of RNA-Seq data and qRT-PCR validation. Sci. Rep..

[58] Barmeyer C., Amasheh S., Tavalali S., Mankertz J., Zeitz M., Fromm M., Schulzke J.-D. (2004). IL-1β and TNFα regulate sodium absorption in rat distal colon. Biochem. Biophys. Res. Commun..

[59] Al-Ghadban S., Kaissi S., Homaidan F.R., Naim H.Y., El-Sabban M.E. (2016). Cross-talk between intestinal epithelial cells and immune cells in inflammatory bowel disease. Sci. Rep..

[60] Sugi K., Musch M.W., Chang E.B., Field M. (2001). Inhibition of Na^+^, K^+^-ATPase by interferon γ down-regulates intestinal epithelial transport and barrier function. Gastroenterology.

[61] Rocha F., Musch M.W., Lishanskiy L., Bookstein C., Sugi K., Xie Y., Chang E.B. (2001). IFN-γ downregulates expression of Na^+^/H^+^ exchangers NHE2 and NHE3 in rat intestine and human Caco-2/bbe cells. Am. J. Physiol.-Cell Physiol..

[62] Howe K., Gauldie J., McKay D.M. (2002). TGF-β effects on epithelial ion transport and barrier: Reduced Cl^−^ secretion blocked by a p38 MAPK inhibitor. Am. J. Physiol.-Cell Physiol..

[63] Greenwood-Van Meerveld B., Tyler K., Keith J.C. (2000). Recombinant human interleukin-11 modulates ion transport and mucosal inflammation in the small intestine and colon. Lab. Investig..

[64] Cross R.K., Wilson K.T. (2003). Nitric oxide in inflammatory bowel disease. Inflamm. Bowel Dis..

[65] Kumar A., Chatterjee I., Gujral T., Alakkam A., Coffing H., Anbazhagan A.N., Borthakur A., Saksena S., Gill R.K., Alrefai W.A. (2017). Activation of Nuclear Factor-κB by Tumor Necrosis Factor in Intestinal Epithelial Cells and Mouse Intestinal Epithelia Reduces Expression of the Chloride Transporter SLC26A3. Gastroenterology.

[66] Manoharan P., Coon S., Baseler W., Sundaram S., Kekuda R., Sundaram U. (2013). Prostaglandins, not the leukotrienes, regulate Cl^−^/HCO_3_^−^ exchange (DRA, SLC26A3) in villus cells in the chronically inflamed rabbit ileum. Biochim. Biophys. Acta (BBA)-Biomembr..

[67] Arthur S., Palaniappan B., Afroz S., Sundaram U. (2021). Unique Regulation of Coupled NaCl Absorption by Inducible Nitric Oxide in a Spontaneous SAMP1/YitFc Mouse Model of Chronic Intestinal Inflammation. Inflamm. Bowel Dis..

[68] Rahman M.M., Borthakur A., Afroz S., Arthur S., Sundaram U. (2021). Unique Regulation of Intestinal Villus Epithelial Cl^−^/HCO_3_^−^ Exchange by Cyclooxygenase Pathway Metabolites of Arachidonic Acid in a Mouse Model of Spontaneous Ileitis. Int. J. Mol. Sci..

[69] Rahman M.M., Afroz S., Arthur S., Sundaram U. (2021). Mast cell mediated regulation of small intestinal chloride malabsorption in SAMP1/YitFc mouse model of spontaneous chronic Ileitis. Cells.

[70] Ding X., Li D., Li M., Wang H., He Q., Wang Y., Yu H., Tian D., Yu Q. (2018). SLC26A3 (DRA) prevents TNF-alpha-induced barrier dysfunction and dextran sulfate sodium-induced acute colitis. Lab. Investig. J. Tech. Methods Pathol..

[71] Saksena S., Singla A., Goyal S., Katyal S., Bansal N., Gill R.K., Alrefai W.A., Ramaswamy K., Dudeja P.K. (2010). Mechanisms of transcriptional modulation of the human anion exchanger SLC26A3 gene expression by IFN-γ. Am. J. Physiol.-Gastrointest. Liver Physiol..

[72] Priyamvada S., Anbazhagan A.N., Gujral T., Borthakur A., Saksena S., Gill R.K., Alrefai W.A., Dudeja P.K. (2015). All-trans-retinoic acid increases SLC26A3 DRA (down-regulated in adenoma) expression in intestinal epithelial cells via HNF-1β. J. Biol. Chem..

[73] Van der Goten J., Vanhove W., Lemaire K., Van Lommel L., Machiels K., Wollants W.-J., De Preter V., De Hertogh G., Ferrante M., Van Assche G. (2014). Integrated miRNA and mRNA expression profiling in inflamed colon of patients with ulcerative colitis. PLoS ONE.

[74] Anbazhagan A.N., Priyamvada S., Kumar A., Maher D.B., Borthakur A., Alrefai W.A., Malakooti J., Kwon J.H., Dudeja P.K. (2014). Translational repression of SLC26A3 by miR-494 in intestinal epithelial cells. Am. J. Physiol.-Gastrointest. Liver Physiol..

[75] Schmidt F.M., Weschenfelder J., Sander C., Minkwitz J., Thormann J., Chittka T., Mergl R., Kirkby K.C., Faßhauer M., Stumvoll M. (2015). Inflammatory cytokines in general and central obesity and modulating effects of physical activity. PLoS ONE.

[76] Winer D.A., Luck H., Tsai S., Winer S. (2016). The intestinal immune system in obesity and insulin resistance. Cell Metab..

[77] Shemtov S.J., Emani R., Bielska O., Covarrubias A.J., Verdin E., Andersen J.K., Winer D.A. (2023). The intestinal immune system and gut barrier function in obesity and ageing. FEBS J..

[78] Khan S., Luck H., Winer S., Winer D.A. (2021). Emerging concepts in intestinal immune control of obesity-related metabolic disease. Nat. Commun..

[79] Kaur K.K., Allahbadia G., Singh M. (2020). Intestinal Immune System in the Regulation of Obesity and Metabolic Syndrome-Therapeutic Implications-A Systematic Review. EC Clin. Exp. Anat..

[80] Spyrou N., Vallianou N., Kadillari J., Dalamaga M. (2021). The interplay of obesity, gut microbiome and diet in the immune check point inhibitors therapy era. Semin. Cancer Biol..

[81] Acciarino A., Diwakarla S., Handreck J., Bergola C., Sahakian L., McQuade R.M. (2023). The role of the gastrointestinal barrier in obesity-associated systemic inflammation. Obes. Rev..

[82] Yde J., Keely S., Wu Q., Borg J.F., Lajczak N., O’Dwyer A., Dalsgaard P., Fenton R.A., Moeller H.B. (2016). Characterization of AQPs in mouse, rat, and human colon and their selective regulation by bile acids. Front. Nutr..

[83] Sundaram U., Wisel S., Rajendren V., West A. (1997). Mechanism of inhibition of Na+-glucose cotransport in the chronically inflamed rabbit ileum. Am. J. Physiol.-Gastrointest. Liver Physiol..

[84] Sundaram U., West A.B. (1997). Effect of chronic inflammation on electrolyte transport in rabbit ileal villus and crypt cells. Am. J. Physiol. Gastrointest. Liver Physiol..

[85] Palaniappan B., Sundaram S., Arthur S., Afroz S., Sundaram U. (2020). Inducible nitric oxide regulates na-glucose Co-transport in a spontaneous SAMP1/YitFc mouse model of chronic ileitis. Nutrients.

